# Scrapping the Trained Nurse

**Published:** 1920-04-03

**Authors:** 


THE EDITOR'S LETTER-BOX—(*continued*).

### Scrapping the Trained Nurse.

*To the Editor of* The Hospital.

Sir,—I am disappointed that Miss Gladys M. Leigh did not endeavour to contribute something more useful to a subject in which she professes to be profoundly interested, than an attempt to prove inconsistency of attitude on my part. My attitude really matters nothing. But, seeing that attention is called to it, permit me to observe that there is no inconsistency. I hold the view that it is the Minister of Health who has veered. In the earlier literature which issued from his Department, great stress was laid on the potential value of a trained nurse as health visitor. The regulations were then in a fluid condition. A considerable number of people expected this to be followed immediately by a co-operation between the Department and the hospitals, and an arrangement made whereby the extra necessary training might be imparted while the nurse remained resident in hospital.

Nothing of the sort happened, but, instead, Dr. Addison, in collaboration with the Board of Education, has decided on a scheme of recruitment in which the trained nurse cannot participate, save in exceptional cases. I live in the hope that the profound interest of your critical correspondent may extent to the practical furtherance of the claims of the trained nurse, as I have so far regarded Miss Leigh as something more than a mere controversialist.

Yours faithfully,

Ierne.

